# Evaluation of bone formation and membrane degradation in guided bone regeneration using a 4-hexylresorcinol-incorporated silk fabric membrane

**DOI:** 10.1186/s40902-015-0034-0

**Published:** 2015-09-30

**Authors:** Sang-Woon Lee, In Chul Um, Seong-Gon Kim, Min-Sang Cha

**Affiliations:** 1 Department of Dentistry, Gangneung Asan Hospital, University of Ulsan College of Medicine, 38, Bangdong-gil, Sacheon-myeon, Gangneung-si, Gangwon-do South Korea; 2 Department of Bio-fibers and Materials Science, Kyungpook National University, Daegu, South Korea; 3 Department of Oral and Maxillofacial Surgery, College of Dentistry, Gangneung-Wonju National University, Gangneung-si, South Korea

## Abstract

**Background:**

The aims of present study were (1) to evaluate new bone formation among the 4-hexylresorcinol (4HR)-incorporated silk fabric membrane (SFM), conventional SFM, and uncovered control groups and (2) to compare the amount of residual membrane between the 4HR-incorporated SFM and conventional SFM in a rabbit parietal defect model.

**Methods:**

Nine New Zealand white rabbits were used for this animal study. After the formation of a bilateral parietal bone defect (diameter 8.0 mm), either 4HR-incorporated SFM or conventional SFM was grafted into the defect. The defect in the control was left uncovered. New bone formation and the amount of residual membrane were evaluated by histomorphometry at 8 weeks after the operation.

**Results:**

The total amount of new bone was 37.84 ± 8.30 % in the control, 56.64 ± 15.74 % in the 4HR-incorporated SFM group, and 53.35 ± 10.52 % in the conventional SFM group 8 weeks after the operation. The differences were significant between the control and 4HR-incorporated SFM group (*P* = 0.016) and between the control and conventional SFM group (*P* = 0.040). The residual membrane was 75.08 ± 10.52 % in the 4HR-incorporated SFM group and 92.23 ± 5.46 % in the conventional SFM group 8 weeks after the operation. The difference was significant (*P* = 0.039).

**Conclusions:**

The 4HR-incorporated SFM and conventional SFM groups showed more bone regeneration than the control group. The incorporated 4HR accelerated the partial degradation of the silk fabric membrane in a rabbit parietal defect model 8 weeks after the operation.

## Background

The main goal of using a barrier membrane in guided bone regeneration (GBR) is to maintain a space for future bone regeneration [1]. In recent decades, absorbable collagen membranes or non-absorbable ePTFE membranes have been used for GBR in dental practice [1, 2]. Although these commercial membranes have been shown to be effective in bone regeneration, their high cost inhibits their widespread clinical application.

Producing a barrier membrane from silk fiber would be advantageous with respect to cost [2]. Previous studies using silk-based barrier membranes evaluated new bone formation after their application in animal models. The silk fibroin film produced by the casting method resulted in greater bone regeneration compared to uncovered controls [3, 4]. The silkworm cocoon membrane produced by simple separation also showed greater amounts of bone regeneration compared to the ePTFE membrane [5].

However, silk-based barrier membranes have some limitations. First, they are non-absorbable. Second, surgery for the removal of the membrane is needed after adequate bone formation. Previous studies showed that the silk fibroin film exhibits high fragility and poor operability during the operation [3, 4]. The silkworm cocoon membrane showed better bone formation compared to the ePTFE membrane [5]. However, the mechanical separation of the silkworm cocoon may be labor intensive and difficult to automate.

In the present study, the silk fabric membrane (SFM) was tested as another silk-based barrier membrane in the animal model. The SFM is produced by a textile manufacturing method. The antiseptic agent 4-hexylresorcinol (4HR) was also incorporated into the SFM for drug release [4]. 4HR has been used as a component of sore throat lozenges [6]. A previous study reported that 4HR may accelerate the degradation of the silk fibroin graft by increasing the activation of macrophages [7]. We thus hypothesized that the degradation of the SFM would be accelerated by the release of 4HR.

The aims of present study were (1) to evaluate new bone formation among the 4HR-incorporated SFM, conventional SFM, and uncovered control groups and (2) to compare the amount of residual membrane between the 4HR-incorporated SFM and conventional SFM in a rabbit parietal defect model.

## Methods

### Silk fabric membrane

The silk fabric membrane (SFM) was kindly provided by Sanju Myungju Co (Sangju, Korea). The silk fabric is the plain weave with a waft density of 45 yarns/inch and a weft density of 47 yarns/inch. The waft yarn is a 140-denier silk filament, and the weft yarn is a twisted yarn from 21d to 25d silk filaments. This silk fabric membrane was degummed to remove sericin before the experiment. The crystallinity of silk fabric is 55.7 % calculated from Fourier transform infrared spectroscopy measurement result. The average pore size of silk fabric membrane is 12,792 μm^2^ determined by digital optical microscope (Toolis, Daegu, Korea).

The SFM was prepared with 10 mm in length and 10 mm in width for animal experiment. The thickness of SFM was approximately 0.3 mm in dry condition and 0.5 mm in wet condition.

### Animals and surgical procedures

**Fig. 1:**
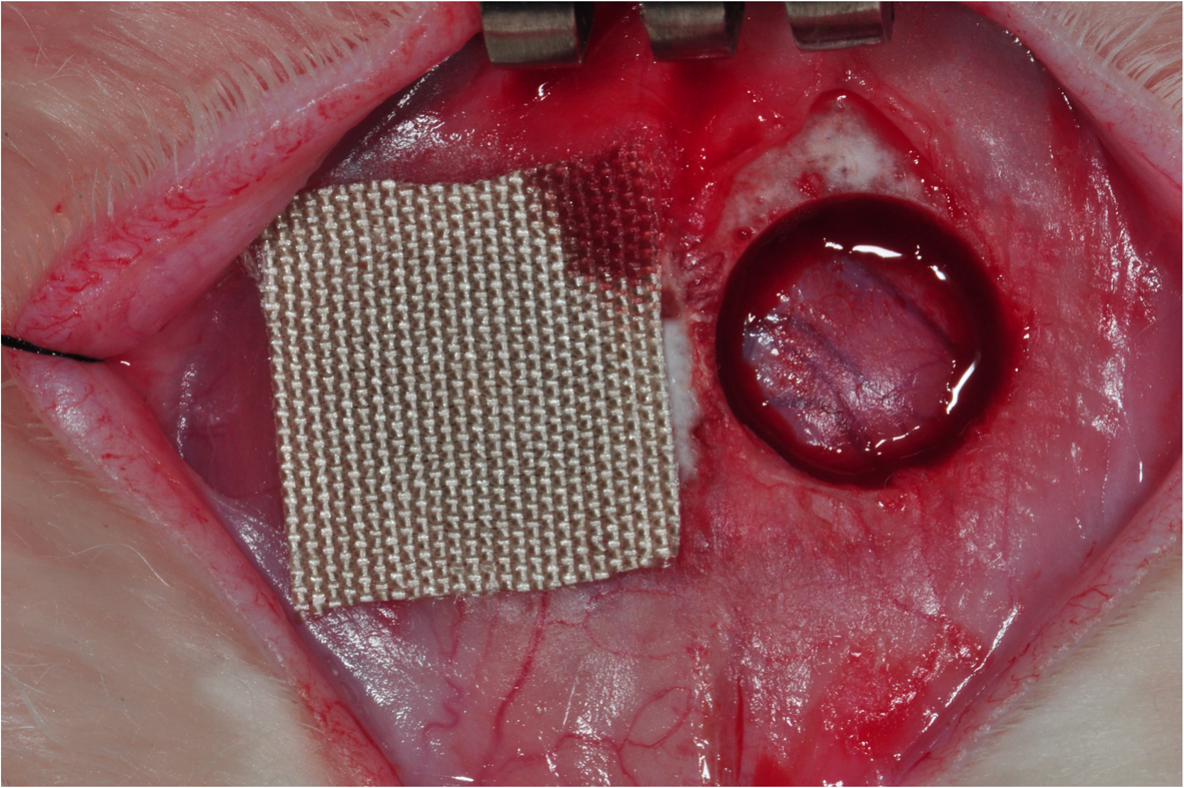
Bilateral parietal bone defect of rabbits. The *right side* contained 4HR-incorporated SFM, and the *left side* served as the uncovered control. *4HR* 4-hexylresorcinol, *SFM* silk fabric membrane

Nine 10-week-old New Zealand white rabbits were used in this experiment, which was approved by the Institutional Animal Care and Use Committee of Gangneung-Wonju National University, Gangneung, Korea (IACUC GWNU- 2014–15). General anesthesia was induced by intramuscular injection of a combination of 0.5 mL of tiletamine and zolazepam (125 mg/mL; Zoletil; Bayer Korea, Seoul, Korea) and 0.5 mL of xylazine hydrochloride (10 mg/kg body weight; Rompun; Bayer Korea). The cranium area was shaved and disinfected with povidine-iodine. A longitudinal incision was made on the midline of the cranium area. Sharp subperiosteal dissection was performed to expose the parietal bones. A dental trephine bur was used under saline irrigation to create a bilateral full-thickness calvarial defect. Two defects 8 mm in diameter were created, one on each side of the midline. Either the 4HR-incorporated SFM or the conventional SFM was placed on the calvarial defects. Some defects remained uncovered and served as the control (Fig. 1). Assignment to each group for the corresponding defect was performed randomly, and each group was composed of six animals (six defects for each group). None of the animals received the same membrane in both calvarial defects. Then, the pericranium and skin were closed in layers with 3-0 black silk. Each rabbit was individually caged and received food and water. Nine animals were sacrificed at 8 weeks after the operation.

### Hematoxylin and eosin staining

The bone samples were decalcified using 5 % nitric acid for 48 h. The right and left parietal bones were separated through the midline sagittal suture. Both segments were embedded to show the sagittal sections in the paraffin blocks. Then, the sections were sliced and stained with hematoxylin and eosin (H&E).

Paraffin-embedded tissue blocks were sliced to a thickness of 5 μm. The sections of each tissue were carefully placed on silane-coated slides. These slides were incubated at 60 °C for 24 h. After cooling at room temperature, the tissue slides were soaked in 100 % xylene for 5 min in triplicate. The tissue sections were then hydrated by the consecutive application of high- to low-grade ethyl alcohol. Fully hydrated tissue sections were washed with distilled water. After that, tissue sections were stained with Harris modified hematoxylin solution (Sigma Aldrich, St. Louis, MO, USA) for 10 min at room temperature. Then, de-staining was performed with 1 % acid alcohol for 1 s. De-stained tissue sections were washed in running tap water for 10 min. Next, Eosin Y solution (Sigma Aldrich, St. Louis, MO, USA) was applied on the tissue sections for 1 min. Then, after gradational hydration with ethyl alcohol and clearing with xylene, the tissue sections were fixed by paramount solutions.

### Histomorphometric evaluation

The sagittal section showing the widest defect area was selected. Digital images of the selected sections were taken using a digital camera (DP-20; Olympus, Tokyo, Japan). The images were analyzed by SigmaScan Pro 5.0; SPSS Science, Chicago, IL, USA). The total amount of new bone was calculated as a percentage of the total region of the defect. The residual membrane was also calculated as a percentage of the residual membrane area 8 weeks postoperatively compared to the original area of membrane.

### Statistical analysis

An ANOVA test was used for comparison of new bone formation of the three groups, and the LSD method was used as a post hoc test. An independent-samples *t* test was used for the comparison of the residual membrane of the two groups. Statistical significance was set at *P* < 0.05.

## Results

The histomorphometry results are presented in Table 1. Total new bone was 37.84 ± 8.30 % in the control group, 56.64 ± 15.74 % in the 4HR-incorporated SFM group, and 53.35 ± 10.52 % in the conventional SFM group 8 weeks after the operation. The differences were significant between the control and 4HR incorporated SFM group (*P* = 0.016) and the control and conventional SFM group (*P* = 0.040).

**Table 1:** Histomorphometric analysis

	Control	4HR incorporated SFM	Conventional SFM
Total new bone (%)	37.84 ± 8.30	56.64 ± 15.74^*^	53.35 ± 10.52
Residual membrane (%)		75.08 ± 10.52	92.23 ± 5.46

*4HR* 4-hexylresorcinol, *SFM* silk fabric membrane

^*^ *P* < 0.05 compared to uncovered control

**Fig. 2:**
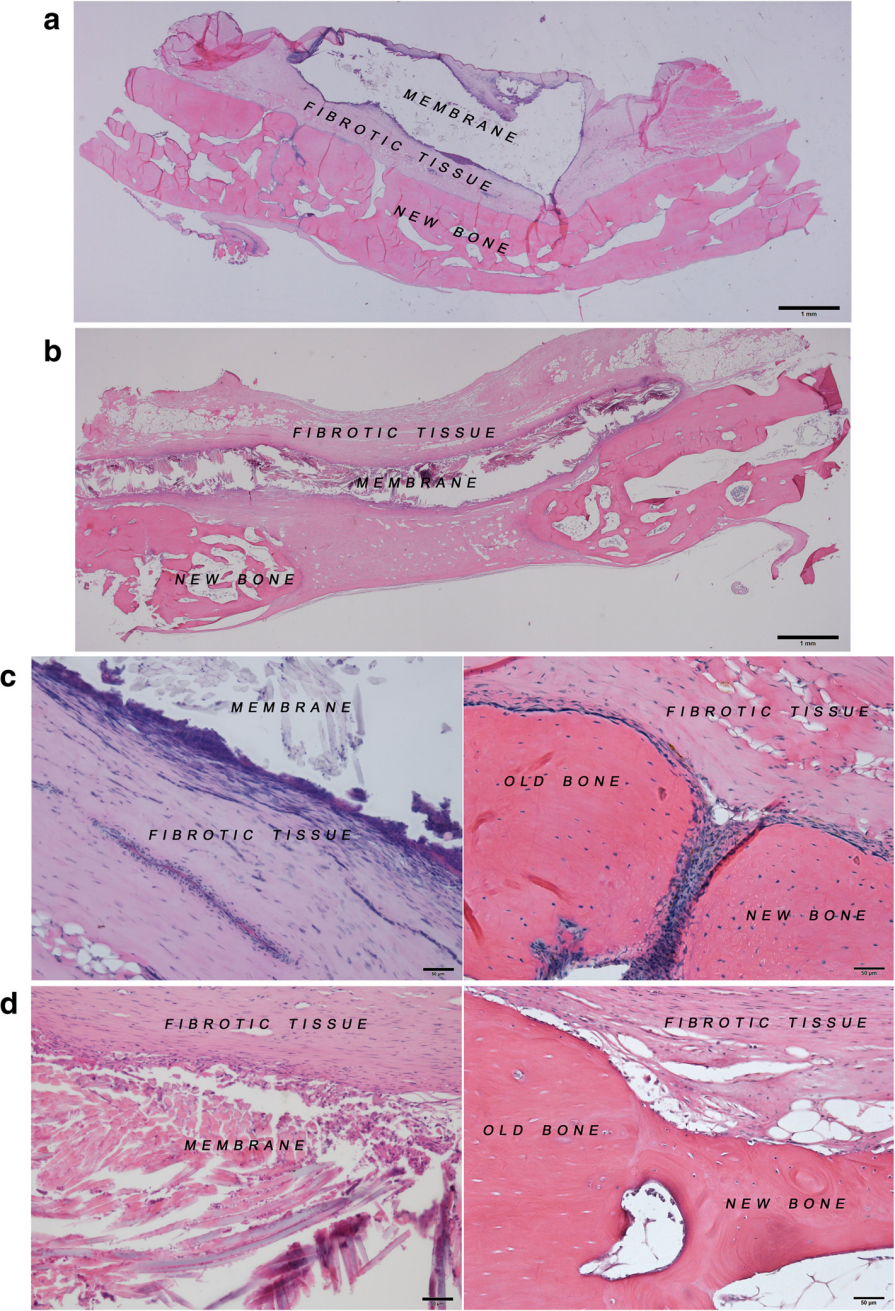
Histological images at 8 weeks after the operation. **a** 4HR-incorporated SFM (*bar* = 1 mm). **b** Conventional SFM (*bar* = 1 mm). **c** 4HR-incorporated SFM (*bar* = 50 μm). **d** Conventional SFM (*bar* = 50 μm)

The residual membrane was 75.08 ± 10.52 % in the 4HR-incorporated SFM group and 92.23 ± 5.46 % in the conventional SFM group at 8 weeks after the operation. The difference was significant (*P* = 0.039). The SFM was encapsulated by thick fibrotic tissue in the SFM group (Fig. 2). The thickness of the SFM in the conventional SFM group was approximately 0.5 mm, almost the same as in the original dimension of the SFM. However, the thickness of the SFM in the 4HR-incorporated SFM group was approximately 0.2 mm.

## Discussion

Silk fiber is composed of fibroin and sericin. Silk fibroin is a main protein that shows biocompatibility and a low immune response in the human body [8]. Silk sericin is a gummy protein that surrounds the silk fibroin. It has been considered to be a biocompatible material; however, immune and irritation reactions to silk sericin have been reported [9, 10]. The SFM in present study, silk sericin was removed by degumming process.

The conventional SFM showed a soft and smooth texture with ivory color (Fig. 1). The main advantage of the conventional SFM is that it is possible to mass-produce with uniform quality and low cost. In clinical application, it can be applied on small- to large-sized bone defects, and the shaping of the membrane can be easily performed by a scissor. The disadvantages of the conventional SFM are that it has low rigidity and is non-absorbable because of high molecular weight and crystallinity of natural silk material; thus, a second surgery for removal is needed.

In the present study, the 4HR-incorporated SFM group and the conventional SFM group showed higher bone regeneration compared to uncovered controls (*P* = 0.016 and *P* = 0.040, Table 1). These results are consistent with previous studies concerning the use of other types of silk-based barrier membranes [3, 4]. The conventional SFM is used as a barrier membrane to prevent soft tissue ingrowth during bone regeneration. When comparing the 4HR-incorporated SFM and the conventional SFM groups, there was no significant difference (*P* > 0.05).

The main advantage of the use of collagen membranes is its gradual resorption during bone regeneration [11]. If the addition of 4HR can lead to complete resorption of the silk membrane, a second surgery for membrane removal is not needed, as in the case of collagen membranes. On the contrary, silk is mostly considered to be a non-absorbable material [12]. However, some studies have reported that the degradation of silk fibroin may be possible after a long period of time in vivo [9, 13]. Silk fibroin can be slowly degraded by proteolytic enzymes in vivo and vitro [9, 14–16]. Macrophages have an important role in the phagocytosis of foreign particles and in the release of proteolytic enzymes [17]. 4HR may increase macrophage activation through the suppression of foreign body giant cell formation in the silk fibroin graft [7].

In this study, the amount of residual membrane was higher in the conventional SFM group compared to 4HR-incorporated SFM group (*P* = 0.039, Fig. 2). This means that the addition of 4HR accelerates the partial degradation of the SFM. The SFM should be fully absorbed in order to be clinically meaningful. However, complete resorption of the SFM was not observed in the present study. Further studies with longer healing periods and different concentrations of 4HR are needed to evaluate the effect of 4HR with respect to the degradation of silk fibroin.

## Conclusions

The SFM groups showed more bone regeneration than the uncovered control group. The incorporated 4HR accelerated the partial degradation of the silk fabric membrane in a rabbit parietal defect model 8 weeks after the operation.

## Abbreviations

4HR: 4-hexylresorcinol
ePTFE: expanded polytetrafluoroethylene
SFM: silk fabric membrane
